# NF-κB Pathway and Its Inhibitors: A Promising Frontier in the Management of Alzheimer’s Disease

**DOI:** 10.3390/biomedicines11092587

**Published:** 2023-09-21

**Authors:** Bhagavathi Sundaram Sivamaruthi, Neha Raghani, Mehul Chorawala, Sankha Bhattacharya, Bhupendra G. Prajapati, Gehan M. Elossaily, Chaiyavat Chaiyasut

**Affiliations:** 1 Office of Research Administration, Chiang Mai University, Chiang Mai 50200, Thailand; sivamaruthi.b@cmu.ac.th; 2 Innovation Center for Holistic Health, Nutraceuticals, and Cosmeceuticals, Faculty of Pharmacy, Chiang Mai University, Chiang Mai 50200, Thailand; 3 Department of Pharmacology and Pharmacy Practice, L. M. College of Pharmacy, Ahmedabad 380009, India; 4 School of Pharmacy & Technology Management, SVKM’S NMIMS Deemed-to-be University, Shirpur 425405, India; 5 Shree S. K. Patel College of Pharmaceutical Education and Research, Ganpat University, Mehsana 384012, India; 6 Department of Basic Medical Sciences, College of Medicine, AlMaarefa University, P.O. Box 71666, Riyadh 11597, Saudi Arabia

**Keywords:** Alzheimer’s disease, NF-κB, amyloid beta plaques, neuroinflammation, phytochemicals

## Abstract

The nuclear factor kappa B (NF-κB) pathway has emerged as a pivotal player in the pathogenesis of various diseases, including neurodegenerative illnesses like Alzheimer’s disease (AD). The involvement of the NF-κB pathway in immune system responses, inflammation, oxidative stress, and neuronal survival highlights its significance in AD progression. We discuss the advantages of NF-κB pathway inhibition, including the potential to mitigate neuroinflammation, modulate amyloid beta (Aβ) production, and promote neuronal survival. However, we also acknowledge the limitations and challenges associated with this approach. Balancing the fine line between dampening inflammation and preserving physiological immune responses is critical to avoid unintended consequences. This review combines current knowledge on the NF-κB pathway’s intricate involvement in AD pathogenesis, emphasizing its potential as a therapeutic target. By evaluating both advantages and limitations, we provide a holistic view of the feasibility and challenges of NF-κB pathway modulation in AD treatment. As the quest for effective AD therapies continues, an in-depth understanding of the NF-κB pathway’s multifaceted roles will guide the development of targeted interventions with the potential to improve AD management.

## 1. Introduction

Alzheimer’s disease (AD), a neurodegenerative disease, is the most common type of dementia, responsible for up to 75% of all cases [1]. It manifests as a clinical syndrome presenting symptoms like impairment of memory, language, and other cognitive functions, and behavioral changes that severely affect the activities of daily living. The worldwide prevalence of dementia is thought to be around 3.9%, which is estimated to escalate with the growing size of the population aged 65 years and older. The fundamental histopathological feature of AD has been considered to be aggregates of amyloid beta (Aβ) plaques in the cortical and limbic regions of the brain [2]. Despite offering a comprehensive framework for understanding AD pathogenesis, certain observations do not seamlessly align with the most straightforward rendition of the hypothesis, leading to its existing lack of intricate explanations [3,4,5]. It is noteworthy that, intriguingly, no treatment closely tied to the proposed amyloid hypothesis has achieved success thus far. Thus, ineffective attempts to manage the progressive symptoms have compelled new insights into pathogenesis. Post-translational modifications (PTMs) are pivotal in AD as they influence the aggregation of pathogenic proteins implicated in the disease [6]. Aberrant PTMs, such as the hyperphosphorylation of tau protein and Aβ, glycation, nitration, and ubiquitination, contribute to the misfolding and aggregation of these proteins. The phosphorylation of tau disrupts its normal function, leading to neurofibrillary tangles (NFTs). Aβ, through PTMs like glycation and nitration, adopts toxic conformations, forming amyloid plaques. Ubiquitination may impair the clearance of misfolded proteins. These PTMs disrupt protein homeostasis, impair cellular functions, and trigger neuroinflammation. The interplay between PTMs and nuclear factor kappa B (NF-κB)-driven inflammation thus contributes to protein aggregation, neurodegeneration, and AD progression.

**Figure 1:**
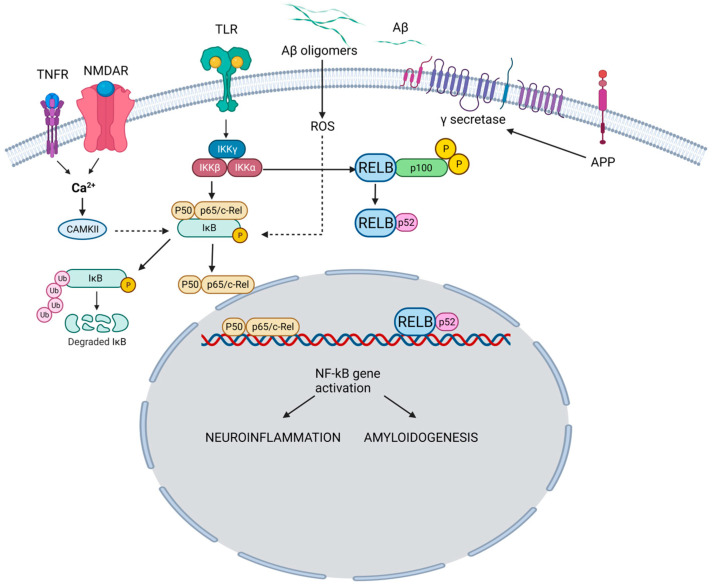
NF-κB pathway and its role in the pathogenesis of AD. In the canonical pathway, TNFR and NMDAR activation raises Ca^2+^, triggering CaMKII-mediated Ca^2+^-NF-κB linkage. Simultaneously, TLR activation acts on IKK subunits. Ca^2+^ leads to IκB kinase activation, IκB breakdown, and p65/p50 dimer formation. NF-κB enters the nucleus, binding neuronal gene targets (e.g., APP) for synaptic function, memory, and amyloid processing. Aβ, resulting from APP cleavage, activates ROS-dependent NF-κB, intensifying amyloid dysregulation. Pro-inflammatory agents (e.g., TNF-α, IL-1β) activate TLR through canonical (IKKβ-NEMO) and non-canonical (IKKγ, IKK-α) pathways. Both drive NF-κB-mediated neuroinflammation and neurodegeneration in AD. TNFR: Tumor necrosis factor receptor; NMDAR: N-Methyl-D-Aspartate Receptor; TLR: Toll-like receptor; Aβ: Amyloid beta; APP: Amyloid precursor protein; CAMKII: Calcium–calmodulin (CaM)-dependent protein kinase I; IKKγ: Inhibitor of nuclear factor-ĸB Kinase gamma (also known as NEMO: Nuclear factor-κB (NF-κB) essential modulator); IKKα: Inhibitor of nuclear factor-ĸB Kinase alpha; IKKβ: Inhibitor of nuclear factor-ĸB kinase beta; IĸB: Inhibitor of ĸB; ROS: Reactive oxygen species. TNF-α: Tumor necrosis factor-alpha, IL-1β: Interlukin-1 beta; RELB: Transcription factor.

The NF-κB family is a group of transcription factors that play a pivotal role in regulating various biological processes, including immune responses, inflammation, cell survival, and cellular differentiation [7]. The family encompasses five members. NF-κB1 regulates immune responses and inflammation (Figure 1).

Upon proteolytic processing, NF-κB1 generates the active p50 subunit. Similarly, NF-κB2 undergoes proteolytic processing to produce the active p52 subunit, which is involved in immune responses and lymphoid organ development. The p65 subunit aids in functional NF-κB dimer formation and is pivotal in regulating inflammation, immune responses, and cellular survival. The transcription factor, RelB, contributes to NF-κB dimer assembly and is particularly associated with immune responses, especially in lymphoid organ development. The c-Rel subunit modulates immune responses and gene expression related to inflammation and lymphoid organ development. These NF-κB family members form homo- or heterodimers, dictating target gene specificity and ensuing biological impacts [8].

Within neuroinflammation, NF-κB occupies a central role in coordinating the expression of genes that contribute to both protective and detrimental aspects of the inflammatory response in the nervous system. Primarily sequestered in an inactive state within the cytoplasm, NF-κB is bound to inhibitory proteins named IκBs. Upon activation, typically induced by a spectrum of stimuli encompassing pro-inflammatory cytokines, pathogens, or cellular stressors, these IκBs undergo phosphorylation and consequent degradation, facilitating the translocation of NF-κB to the nucleus. This nuclear migration empowers NF-κB to engage specific DNA sequences, thereby orchestrating the modulation of an extensive array of target genes. NF-κB signaling also influences the integrity of the blood–brain barrier (BBB), governing the passage of molecules between the bloodstream and the brain milieu. The activation of NF-κB in the endothelial cells of the BBB provokes heightened permeability, allowing immune cells and molecules to enter the brain [9].

Further, NF-κB activation in microglia triggers their transition from resting to activated states, releasing pro-inflammatory cytokines, chemokines, and reactive oxygen species (ROS). NF-κB is pivotal for initiating protective immune responses against pathogens and facilitating tissue repair. However, prolonged NF-κB activation within neurons can result in neurotoxicity, contributing to oxidative stress, excitotoxicity, and inflammation, all of which undermine neuronal well-being. NF-κB also influences the balance between neuronal survival and apoptosis. In some cases, NF-κB activation can promote the expression of anti-apoptotic genes, supporting neuronal survival [10]. However, chronic NF-κB activation can lead to a pro-apoptotic response in other situations.

This review summarizes the role of the NF-ĸB pathway as a potential target in treating AD, along with the prospects and challenges of its modulation as a therapeutic strategy. Also, the manuscript discusses the interplay between inflammation and neurodegeneration.

## 2. Role of NF-κB Pathway Inhibitors in AD Treatment

The NF-κB pathway plays a significant role in neuroinflammation and other processes implicated in AD pathogenesis. Consequently, it has been considered a potential therapeutic target for the management of AD [11]. However, targeting the NF-κB pathway is complex due to its multifaceted role in promoting and regulating inflammation and immune responses. NF-κB pathway inhibitors offer the means to modulate this intricate signaling network and counteract neuroinflammation. By inhibiting NF-κB activation, these inhibitors can dampen the excessive release of pro-inflammatory molecules, thereby reducing the harmful impact of inflammation on neurons [12]. They hold the potential to alleviate neuronal damage and promote their survival in the face of AD-associated challenges.

Furthermore, NF-κB inhibitors may influence key pathological hallmarks of AD: Aβ plaques and tau tangles. NF-κB activation regulates enzymes involved in Aβ production and clearance [13]. Inhibiting this pathway could potentially modify Aβ metabolism, impacting the deposition and accumulation of Aβ plaques. Similarly, NF-κB activation has been linked to tau hyperphosphorylation and tangle formation. Inhibitors might intervene in these processes, attenuating tau pathology and promoting neuronal stability. However, it is essential to tread cautiously in inhibiting NF-κB, as this pathway also plays crucial roles in immune responses and cell survival [14]. A complete blockade could compromise the immune system’s ability to defend against pathogens and maintain tissue integrity. Striking the right balance between dampening neuroinflammation and preserving necessary immune functions is critical. The direct inhibition of NF-κB activation is a strategy under investigation for its therapeutic potential in various diseases, including AD [15]. Directly inhibiting NF-κB activation modulates the aberrant inflammatory response, potentially slowing disease progression. Researchers are exploring small molecules and compounds that directly target components of the NF-κB signaling pathway. These agents can interfere with the activation steps that lead to NF-κB translocation into the nucleus, where it regulates gene expression.

Advancements in NF-κB pathway inhibitor research are multifaceted. Scientists are striving to develop selective inhibitors that target specific pathway components, minimizing off-target effects. A study conducted by Elzayat and colleagues investigates mesenchymal stem cells (MSCs) and acitretin for AD treatment, focusing on the NF-kB pathway and miRNA regulation. Using a rat model, miR-146a, miR-155, necrotic, growth, and inflammatory genes were analyzed. Results showed that MSCs and acitretin restored normal levels, indicating potential therapeutic benefits. MiR-146a and miR-155 are proposed as AD biomarkers. This study underscores MSCs and acitretin’s capacity to modulate miRNAs and related genes in the NF-kB pathway, offering insights for future AD interventions [16]. 

Melatonin, a hormone the pineal gland produces, has been recognized for its role in regulating sleep–wake cycles and its potential anti-inflammatory properties. While melatonin’s direct impact on the NF-κB pathway is limited, it has been suggested that melatonin can indirectly modulate NF-κB activation through various mechanisms. Oxidative stress can activate NF-κB, leading to the expression of pro-inflammatory genes. Melatonin’s antioxidant properties indirectly inhibit NF-κB activation by reducing the triggering oxidative signals [17]. Melatonin can influence molecules downstream of the NF-κB pathway. For instance, it has been reported to downregulate COX-2 (Cyclooxygenase-2) expression, an inflammation enzyme that NF-κB often regulates. Melatonin’s neuroprotective effects may indirectly impact NF-κB activation. By preserving neuronal health and reducing neuroinflammation, melatonin can contribute to an environment where NF-κB activation is less pronounced. Merlo et al. demonstrated the counteracting effects of melatonin on the pro-inflammatory properties of lipopolysaccharides in in vitro models [18]. Another study investigated a phosphodiesterase 5 inhibitor (PDE5I) with dual antagonistic action on IKKB and TNFR1 to inhibit NF-kB and neuroinflammation. In silico docking with FDA-approved compounds identified avanafil with optimal IKKB and TNFR1 binding. Molecular dynamic studies confirmed avanafil’s stability with these targets. In a mouse model of lipopolysaccharide (LPS)-induced neuroinflammation and cognitive decline, avanafil at 6 mg/kg improved cognitive performance, reduced Aβ levels, and lowered inflammatory markers and oxidative parameters [19]. 

Bay 11-7082 is a synthetic small molecule that has gained attention for its potential as an NF-κB pathway inhibitor. This compound has shown inhibitory effects on IκB kinase (IKK), allowing NF-κB to translocate into the nucleus and initiate the expression of target genes involved in inflammation and immune responses. By inhibiting IKK and IκB degradation, Bay 11-7082 effectively interferes with interleukin-6 (IL-6) secretion and the subsequent activation of NF-κB, leading to decreased pro-inflammatory gene expression [20]. For Bay 11-7082 to effectively modulate NF-κB activity within the brain, it must cross the BBB. Developing strategies to enhance its delivery and distribution to the central nervous system while minimizing systemic effects is still a significant challenge [18]. Additionally, a low dose of recombinant IL-2 (aldesleukin) is currently under clinical trial for its clinical application in AD (NCT05821153; NCT05468073).

The SN50 analog is a cell-permeable, synthetic peptide derived from the NF-κB nuclear localization sequence, and it holds potential in AD treatment due to its ability to inhibit NF-κB nuclear translocation. A study conducted by Lin and colleagues suggests that the inhibitory effect is not due to disrupting the binding activity of NF-κB complexes but rather its ability to enter the cell and compete with NF-κB complexes for the cellular machinery responsible for nuclear translocation [21,22]. A study by Pogue and team reveals that upregulated miRNA-30b, associated with neuropathology in AD brain and LPS-stressed human neuronal–glial cells, targets neurofilament light (NF-L)-chain mRNA’s 3′-UTR. This results in the post-transcriptional downregulation of NF-L expression, observed in AD and LPS-treated cells, leading to neuronal cytoskeleton atrophy and synaptic disruption. MiRNA-30b is highly expressed in Aβ-treated models, facilitating LPS entry into neurons. Elevated miRNA-30b induces neuro-injury, inflammation, synaptic impairment, and neuron loss. This gut-microbiota-derived LPS-NF-kB-miRNA-30b-NF-L pathway links GI tract microbes’ LPS to AD’s neuropathology and synaptic disruption, offering insights into neurodegeneration mechanisms [23]. Optimizing dosages to achieve the desired therapeutic outcomes while avoiding potential side effects is an ongoing challenge. Table 1 enlists molecules being researched for their potential role in the NF-ĸB pathway in AD.

In practical terms, combining NF-κB inhibition with other therapeutic approaches may yield enhanced results. Research conducted by Shehata et al. aimed to formulate nanostructured lipid carriers (NLCs) that co-encapsulate donepezil and astaxanthin (DPL/AST–NLCs) and assess their effectiveness in AD-like rat models following daily intranasal administration [41]. AD is a complex disease with multiple underlying mechanisms, including Aβ aggregation, tau pathology, and synaptic dysfunction. Integrating NF-κB inhibitors with interventions targeting these aspects could offer a comprehensive treatment strategy, potentially addressing symptom relief and disease modification.

To translate NF-κB inhibition into clinical practice, robust clinical trials are imperative. These trials should involve diverse patient populations, incorporate appropriate outcome measures, and assess long-term safety. Furthermore, identifying reliable biomarkers that reflect NF-κB activity and its downstream effects will aid in monitoring treatment efficacy and patient response.

## 3. Factors Affecting NF-κB Inhibitors-Based AD Treatments

Inhibiting NF-κB in the context of AD presents a formidable scientific challenge due to the intricate and multifaceted nature of the disease and the pivotal role of NF-κB in immune responses and inflammation. NF-κB serves a dual role, exhibiting both pro-inflammatory and anti-inflammatory functions. It plays a crucial part in the innate immune response, essential for pathogen clearance and tissue homeostasis. For instance, NF-κB activation in microglial cells can release pro-inflammatory cytokines, such as TNF-α and IL-1β, contributing to neuroinflammation in AD [42]. However, NF-κB also has anti-inflammatory functions by promoting the expression of anti-inflammatory cytokines like IL-10. Thus, the complete inhibition of NF-κB may risk suppressing these protective anti-inflammatory responses, vital for maintaining brain homeostasis [43].

Consequently, attempting to completely inhibit NF-κB raises concerns regarding impaired immune responses, necessitating a delicate balance between mitigating its detrimental effects in AD and preserving its protective capabilities. One instance highlighting this challenge is that a non-specific inhibition of NF-κB could disturb this control mechanism, resulting in unregulated NF-κB activation and consequently harming neurons [44]. Furthermore, NF-κB is rigorously controlled through various mechanisms, including inhibitory proteins (IκBs) and post-translational modifications, making precise and specific inhibition challenging. NF-κB operates within diverse cell types in the central nervous system, including neurons and glial cells, with its effects contingent upon cell type, rendering it problematic to target NF-κB without unintentional consequences in various cell populations. In astrocytes, NF-κB can have protective effects by regulating the release of neurotrophic factors. These beneficial effects might be compromised if NF-κB is inhibited without cell type specificity [45]. 

Additionally, AD is a progressive ailment encompassing distinct stages, and the contribution of NF-κB may evolve throughout the disease course, necessitating the consideration of temporal factors for therapeutic intervention. Furthermore, the limited capacity of numerous potential NF-κB inhibitors to penetrate the blood–brain barrier (BBB) effectively poses a significant challenge, as inhibitors must adequately reach neural tissues to modulate NF-κB activity. The potential for off-target effects, impacting alternate signaling pathways or cellular functions, further complicates NF-κB inhibition. Moreover, AD’s heterogeneity, characterized by diverse underlying mechanisms and pathological manifestations, results in NF-κB potentially exerting differential impacts on disease progression among individuals, augmenting the complexity of targeted inhibition. Furthermore, the intricate web of interactions between NF-κB and other signaling pathways, including those associated with amyloid-beta and tau pathology, further complicates the potential outcomes of NF-κB inhibition. Inhibiting NF-κB, for example, may disrupt its interaction with the vital Notch signaling pathway, with potential consequences for neuronal development and function, thereby impacting neuronal health [46]. Lastly, the inhibition of NF-κB may trigger adaptive or compensatory cellular responses, potentially leading to the activation of alternative pro-inflammatory pathways, posing additional challenges in achieving effective and durable therapeutic outcomes [47].

## 4. Insights and Challenges to Targeting the NF-κB Pathway

In the context of AD, the inhibition of NF-κB holds substantial promise as a therapeutic target. As a central regulator of neuroinflammation, NF-κB inhibition can mitigate the excessive inflammatory responses witnessed in AD brains, potentially alleviating the harmful consequences of chronic inflammation on neuronal health. By influencing Aβ metabolism and accumulation, the engagement of NF-κB establishes the possibility of utilizing its inhibition to regulate Aβ production and clearance [48]. This modulation could potentially mitigate Aβ buildup and delay the development of plaques. The intricate role of NF-κB in neuronal survival is noteworthy; it can induce the expression of anti-apoptotic genes, thereby offering potential neuroprotective effects upon inhibition [49]. Notably, targeting NF-κB addresses symptomatic manifestations and certain fundamental mechanisms underpinning AD pathogenesis, suggesting a capacity to modify disease progression. Integrating NF-κB inhibitors with other therapeutic strategies aimed at various aspects of AD pathology holds promise for comprehensive treatment effects. The wealth of research on NF-κB inhibitors in diverse disease contexts provides a valuable foundation for AD treatment development. Leveraging this existing knowledge accelerates the translational potential of NF-κB inhibition as a viable strategy for AD. The well-established role of NF-κB in AD pathogenesis solidifies its standing as a rational and scientifically grounded therapeutic target, bolstering the credibility of NF-κB inhibition as a plausible avenue for effective AD treatment.

The exploration of NF-κB as a therapeutic target in AD holds significant promise, but several challenges must be addressed for successful clinical applications. Subsequent research should emphasize formulating strategies for the selective modulation of NF-κB activity within distinct cell types, encompassing neurons, microglia, and astrocytes [50]. This precise targeting holds the potential to avert undesired effects while achieving therapeutic objectives. Precisely regulating the extent of NF-κB inhibition stands as a critical imperative. Total abrogation might compromise NF-κB’s advantageous contributions to immune responses and cellular survival [51]. There is a need to devise methodologies that establish an equilibrium between attenuating neuroinflammation and safeguarding fundamental cellular functions. Given the intricate nature of AD, amalgamating therapies focused on NF-κB with complementary interventions could yield enhanced outcomes [52]. 

Integrating NF-κB inhibition with strategies addressing amyloid-beta accumulation, tau pathology, or synaptic dysfunction could yield a holistic therapeutic approach. Acknowledging the heterogeneous nature of AD patients and the diverse mechanisms underlying the ailment, prospective treatments could necessitate customization based on individual molecular profiles and disease progression [53,54]. Pioneering the development of dependable biomarkers reflecting NF-κB activity and downstream ramifications within the brain could facilitate gauging treatment efficacy and patient responsiveness to NF-κB-targeted interventions. Overcoming the considerable challenge of designing therapeutic agents capable of crossing the BBB to access the central nervous system requires concentrated research on enhancing drug delivery methodologies. Safeguarding the long-term safety of NF-κB-targeted therapies holds paramount importance. Prolonged NF-κB inhibition may engender off-target ramifications, potentially influencing immune responses and broader physiological processes [55]. A comprehensive evaluation of safety implications is imperative. Methodical and meticulously designed clinical trials are necessary to systematically assess the effectiveness and safety of NF-κB-targeted therapies. Such trials should incorporate diverse patient cohorts, employ pertinent outcome measures, and delineate well-defined endpoints. The disparate impacts of distinct NF-κB inhibitors on neuroprotection, inflammation, and immune responses necessitate further exploration. A comprehensive investigation is warranted to discern the potential of these inhibitors in addressing specific facets of AD pathogenesis. Beyond alleviating symptomatic manifestations, the trajectory of NF-κB-targeted therapies should encompass the development of interventions capable of arresting or decelerating the inherent progression of the disease, thereby confronting the fundamental causes of AD [42].

## 5. Future Directions

The prospects of NF-κB as an AD therapeutic target lie in refining its modulation to culminate in optimal therapeutic consequences. The convergence of advancements in precision medicine, biomarker elucidation, drug delivery modalities, and an enriched comprehension of NF-κB’s intricate roles in AD pathogenesis is poised to catalyze the successful transition of this strategy from preclinical research to efficacious clinical treatments. 

The future direction of targeting NF-κB inhibition through phytochemicals represents a promising and innovative avenue for AD treatment [56]. This approach capitalizes on the potential of natural compounds derived from plants to modulate NF-κB activity, offering a novel way forward in AD management. Several key directions and strategies can shape the progression of NF-κB inhibition using phytochemicals as a target for AD. Future research should prioritize the identification of phytochemicals with robust NF-κB-inhibitory properties. Screening and evaluating various plant-derived compounds can uncover novel candidates that effectively modulate NF-κB signaling pathways. Curcumin has demonstrated potent anti-inflammatory properties by inhibiting NF-κB activation. It may also impact amyloid-beta aggregation and tau phosphorylation, key factors in AD pathogenesis [57]. Epigallocatechin gallate (EGCG) is a bioactive polyphenol found abundantly in green tea, and it has gained attention for its potential benefits in AD management. EGCG has been shown to suppress the activation of NF-κB, a key regulator of inflammation. By inhibiting NF-κB, EGCG can mitigate the production of pro-inflammatory cytokines, chemokines, and other molecules involved in neuroinflammation, a prominent feature of AD [58]. *Salvia miltiorrhiza* is a traditional Chinese herbal medicine that has garnered attention for its potential benefits in AD management. The primary active compounds (Tanshinones and salvianolic acids) in *S. miltiorrhiza* have been investigated for their multifaceted effects, including their potential interaction with NF-κB. *S. miltiorrhiza* has been explored for its potential to influence Aβ metabolism. By modulating NF-κB, *S. miltiorrhiza*’s compounds might impact Aβ production, clearance, and aggregation, key factors in AD pathogenesis. *S. miltiorrhiza* compounds have shown promise in reducing tau hyperphosphorylation, a key feature of neurofibrillary tangles in AD [59]. 

Ashwagandha (*Withania somnifera*), an adaptogenic herb with a long history in traditional medicine, has gained attention for its potential benefits in AD management. The active compounds in ashwagandha, known as withanolides, have been studied for their diverse effects, including their potential interaction with NF-κB signaling. Ashwagandha has been suggested to enhance the expression of neurotrophic factors that support neuronal growth and plasticity. Its interaction with NF-κB may play a role in these effects. Using a *Drosophila melanogaster* AD model, a study explored Ashwagandha’s impact on beta-amyloid toxicity and longevity. At 20 mg/mL, Ashwagandha rescued AD-related phenotypes and enhanced longevity in AD and wild-type *Drosophila*. These findings suggest Ashwagandha’s potential as a potent AD treatment and cellular well-being enhancer [60]. Turner et al. conducted a 52-week randomized, placebo-controlled study investigating resveratrol’s effects on mild to moderate AD. Resveratrol, well tolerated and able to cross the BBB, showed altered biomarker trajectories. Notably, CSF Aβ40 and plasma Aβ40 levels decreased less in the resveratrol group compared to placebo after 52 weeks. It exhibits antioxidant activity, which can counteract oxidative-stress-induced NF-κB activation. While providing class II evidence for resveratrol’s safety and influence on AD biomarkers, further research is necessary to understand these changes [61]. While *Ginkgo biloba* is generally considered safe for short-term use, its efficacy in treating AD remains debatable [62]. Clinical trials examining the effects of *G. biloba* in AD have yielded mixed results. While some studies have reported modest cognitive improvements and slowed decline, others have found no significant benefit compared to placebo. Table 2 lists the representative phytochemicals demonstrating promising bioactivity against AD encompassing the NF-ĸB signaling pathway. A comprehensive understanding of how specific phytochemicals modulate NF-κB activation is crucial. Research efforts should elucidate the molecular mechanisms underlying their effects, potentially leading to the development of targeted therapies with minimized off-target effects.

## 6. Conclusions

NF-κB emerges as a pivotal influencer over both the onset and progression of AD. As elucidated, the dual nature of NF-κB becomes evident—a guardian of immune responses and cell survival, yet also a potential instigator of neuroinflammation and neuronal compromise. The multifaceted contributions of NF-κB in AD pathogenesis have stimulated investigations into harnessing its potential as a therapeutic target. Efforts to manipulate NF-κB, both in preclinical models and clinical contexts, present a compelling approach to mitigating the inflammatory milieu characteristic of AD. Yet, this endeavor is not devoid of challenges. The intricate balance between dampening neuroinflammation and preserving essential cellular functions underscores the delicate nature of NF-κB modulation. Its intricate interplay with other molecular players, such as β-secretase and amyloid-beta, demands a nuanced understanding to untangle the intricacies of AD pathogenesis. The evolving trajectory of NF-κB-targeted therapies hinges on precision—selectively addressing specific cell types, customizing interventions based on individual profiles, and optimizing drug delivery methodologies. The amalgamation of NF-κB-focused strategies with complementary approaches offers a comprehensive approach to tackling the multifaceted nature of AD. These considerations and the imperative of safety assessments and robust clinical trials aid in developing or improving AD treatments. Further studies are required to understand the potential of the NF-κB pathway and its inhibitors in managing AD.

## Figures and Tables

**Table 1:** The list of representative molecules targeting the NF-κB pathway to treat and manage Alzheimer’s disease.

Molecule	NF-ĸB Target	Experimental Setting	Mechanism of Action	References
Alogliptin	Direct	In vitro	Modulates TLR4/MYD88/NF-κB and miRNA-155/SOCS-1 signaling pathways	[24]
AS62868	Direct	In vitro	Inhibits IKKβ	[25]
Docosahexaenoic acid	Indirect	In vitro	Precursor to produce neuroprotection D1, a neuro-protecting agent in the CNS	[26]
Etanercept	Direct	RCT	TNF-α activity inhibitor	[27,28]
Forsythoside B	Direct	In vivo and in vitro	Reduces the serine 536 phosphorylation of IKKα/β, IκBα, and p65	[29]
Minocycline	Direct	In vivo and in vitro	Reduction in IL-6; BACE inhibition	[30,31]
Pioglitazone	Indirect	In vitro	Downregulates glycogen synthase kinase 3 beta and cyclin-dependent kinase in microglia cells	[32,33]
PUFA-Plasmalogens	Direct	In vitro	Inhibits NF-kB, p38MAPK, and JNK pathways	[34]
Simufilam	Indirect	Inpatient	Reduces mTOR basal overactivity; restores the normal shape and function of Filamin A	[35]
Telmisartan	Indirect	In vivo and in vitro	AT_1_ blocking reduces IL-1β levels, resulting in anti-neuroinflammatory effects through JNK/c-Jun and NADPH oxidase pathways; partial PPAR-gamma-stimulating activity	[36,37,38]
TPCA-1	Direct	In vivo	Inhibits IKKβ	[39]
VX-745	Indirect	In vivo	p38 MAPKα inhibitor	[40]

mTOR: Mammalian target of rapamycin; IKKβ: Nuclear factor kappa B kinase subunit beta; TNF-α: Tumor necrosis factor alpha; IL-6: Interleukin 6; BACE: β-Site amyloid precursor protein-cleaving enzyme; CNS: Central nervous system; NF-kB: Nuclear factor kappa B; p38MAPK: p38 mitogen-activated protein kinases; JNK: c-Jun N-terminal kinase; MYD88: Myeloid differentiation primary response protein 88; miRNA-155: MicroRNA-155; SOCS-1: Suppressor of cytokine signaling 1; IκBα: Nuclear factor of kappa light polypeptide gene enhancer in B-cells inhibitor, alpha; p38 MAPKα: p38 mitogen-activated protein kinase alpha. RCT: Randomized clinical trials; AT_1_: Angiotensin 1; NADPH: Nicotinamide Adenine Dinucleotide Phosphate Hydrogen.

**Table 2:** Phytochemicals targeting the NF-κB pathway as therapeutic agents for Alzheimer’s disease.

Phytochemicals	Origin	Mechanism of Action	Experimental Setting	References
Kai–Xin–San (KXS)	*Radix et Rhizoma Ginseng* *Radix Polygalae,* *Acori tatarinowii Rhizoma, Wolfiporia extensa*	Wnt/β-catenin signaling activation; IRE1/XBP1s pathway inhibition	In vivo	[63]
Scoparone	*Artemisia scoparia*	TLR4/MyD88/TRAF-6/TAK-1/NF-κB axis inactivation	In vivo	[64]
Moxibustion	*Artemisia vulgaris*	Reduces the expression of CD206 and secretion of IL-10; increases microglial polarization (M1 to M2)	In vivo	[65]
Citropten	*Citrus aurantifolia*	MAPK and PLCγ/Ca^2+^ pathway modulation	In vitro	[66]
Diterpenoids (Caeminaxin A, B, and others)	*Caesalpinia minax* Hance	Inhibition of iNOS, COX-2 expression; suppresses phosphorylation of MAPK; suppresses activation of NF-ĸB pathway	In vitro	[67]
Icariin (ICA)	*Epimedium koreanum*	Upregulates PPARγ, TAK1/IKK/NF-κB, and JNK/p38 MAPK signaling pathways; PSD-95 regulation	In vivo	[68]
Andrographolide	*Andrographis paniculata*	Enhances the expression of LRP-1, NF-ĸB; decreases IL-1β, IL-6, and TNF-α levels	In vitro	[69]
Biochanin-A	*Trifolium pretense* *Medicago sativa*	Increases phosphorylation of PI3K and Akt	In vivo	[70,71]
Malvidin-3-O-glucoside (*B. atrocarpa* anthocyanin)	*Berberis atrocarpa* Schneid.	NF-κB, IκB, TLR4, and MyD88 downregulation	In silico, in vitro	[72]
Erjing Pills	*Polygonatum sibiricum* *Lycium chinense*	IL-1β, TNF-α, and IL-6 reduction; down regulated TLR4, p-NF-κB P65/NF-κB P65, p-IκBα/IκBα, and NLRP3 expression levels	In vivo	[73]
Licochalcone A	*Glycyrrhiza glabra*	Decrease in 8-iso-PGF_2α_, IL-6 and TNFα levels	In vitro	[74]
Honokiol	*Magnolia officinalis*	NF-κB inhibition, decrease in Aβ plaques	In vivo	[75]
Shi chang pu (SCP) essential oil	*Acorus tatarinowii* Schott	Inhibits phosphorylation of IKKβ, NF-κB, and NLRP3	In vivo	[76]
Cardamonin (CD)	*Alpinia katsumadai* *Alpinia conchigera*	Decreases TNF-α, IL-1, and IL-6 levels; NF-kB and STAT3 modulation	In vitro	[77,78]
Platycodin D	*Platycodon grandiflorus*	TLR4 and p-p65 expression attenuation; decrease in ROS and MDA production	In vitro	[79]
Baicalin (BA)	*Scutellaria baicalensis*	Blocking signal transduction via TLR4, facilitated by the TLR4/MyD88/NF-κB and MAPK pathways; CX3CR1/NF-κB signaling pathway modulation	In vitro	[80,81]
Isorhamnetin	*Oenanthe javanica*	Downregulation of IBA1, NF-κB and CD11b expression	In vitro	[82]
Esculetin	*Artemisia capillaris* Thunb	Inhibits NO production and iNOS expression; inhibits nuclear translocation of NF-κB p65	In vitro	[83]

Wnt/β-catenin: Wingless-related integration site/β-catenin; IRE1/XBP1s: Inositol-requiring enzyme 1/X-box binding protein 1; TRAF-6: Tumor necrosis factor receptor (TNFR)-associated factor 6; TAK-1: Transforming growth factor-β (TGF-β)-activated kinase 1; NF-κB: Nuclear factor kappa B; CD206: Cluster of differentiation 206; IL-10: Interleukin 10; MAPK: Mitogen-activated protein kinases; PLCγ/Ca^2+^: Phospholipase Cγ/Calcium^2+^; iNOS: Inducible nitric oxide synthase; COX-2: Cyclooxygenase-2; PPARγ: Peroxisome proliferators-activated receptor gamma; IKK: Nuclear factor kappa B kinase; JNK/p38 MAPK: c-Jun N-terminal kinase/p38 mitogen-activated protein kinases; PSD-95: Postsynaptic density protein 95; LRP-1: Low-density lipoprotein (LDL)-related protein-1; IL-1β: Interleukin-1 beta; IL-6: Interleukin 6; TNF-α: Tumor necrosis factor alpha; PI3K: Phosphoinositide 3-kinases; Akt: Protein kinase B; p-IκBα/IκBα: Phosphorylated nuclear factor of kappa light polypeptide gene enhancer in B-cells inhibitor/nuclear factor of kappa light polypeptide gene enhancer in B-cells inhibitor; NLRP3: Nucleotide-binding domain, leucine-rich-containing family, pyrin domain-containing-3; 8-iso-PGF_2α_: 8-iso-Prostaglandin F2α; Aβ plaques: Amyloid beta plaques; IKKβ: Nuclear factor kappa B kinase subunit beta; STAT3: Signal transducers and activators of transcription 3; ROS: Reactive oxygen species; MDA: Malondialdehyde; CX3CR1: C-X3-C Motif chemokine receptor 1; IBA1: Ionized calcium-binding adapter molecule 1; NO: Nitric oxide; iNOS: Inducible nitric oxide synthase.

## Data Availability

No new data were created or analyzed in this study. Data sharing does not apply to this article.
